# Reports on the Hospitals of the United Kingdom

**Published:** 1910-01-29

**Authors:** Henry Burdett


### REPORTS

ON THE

### Hospitals of the United Kingdom.

By SIR HENRY BURDETT, K.C.B., K.C.V.O.

### XXII.—GENERAL INFIRMARY, LEEDS.

The North of England contains much that is of interest to hospital managers, and especially to those who have the responsibility of expending large sums of money upon hospital buildings. At Leeds we have a hospital which attracted great attention as a novel departure in construction when it was opened forty years ago. At Liverpool there is the Royal Infirmary building from the plan of one of the greatest of modern architects, the late Mr. Alfred Waterhouse, R.A., which has been in full working order for twenty years past, and at Manchester and at Newcastle-on-Tyne are two great hospitals, the former containing five hundred beds, and so being the largest of provincial hospitals. A careful inspection of all these institutions will yield many features of interest and instruction for the expert. He is able to see what were the prevailing principles which were thought essential half a century ago, what advances have been made scientifically and administratively three decades later, and what the modern system of limited competition for hospital plans produces in the present day. It is a grave responsibility, and by no means an easy task, to weigh the essential differences, advantages, drawbacks, good and bad points which are illustrated in the plans of the great hospitals we have referred to. Speaking generally, it is, of course, impossible, as it would also be grossly unfair, to criticise the older hospital with the same close scrutiny and the same accurate knowledge of the requirements of a hospital to-day that must properly be applied to the new hospitals opened recently. We make these introductory remarks in order to remove misapprehension and to enable those who are studying these reports to gain from them as much information and knowledge as possible.

The foundation stone of the General Infirmary, Leeds, was laid in March 1864 by Mr. James Kitson, and the buildings were completed and opened for the reception of patients in June 1869. A novel plan for adding to the Building Fund was devised by the organisation of a Fine Art Exhibition in the hospital buildings at Leeds before they were opened, and securing the presence of his Majesty King Edward VII., who was then Prince of Wales, who opened the exhibition.

### Bed Capacity for Semi-Convalescents.

The hospital, when originally opened, contained 328 beds, but an extension building was erected and opened in 1892, so that the beds at the present time are 395, the average number occupied being upwards of 360, with a tendency to increase. The bed accommodation is for practical purposes largely increased by a special arrangement peculiar to this infirmary, which has built at Cookridge, near Leeds, and has under its management, two semi-convalescent hospitals, the Ida and Robert Arthington. These branch hospitals contain 88 beds, which are practically always full. To them are sent patients suffering from open wounds, and the system is found in practice to offer many advantages and to be, in fact, a great boon to the patients. We paid a visit to these institutions and made a careful inspection. They are conducted and managed like a hospital, and have a resident medical officer with a sister in charge and seven nurses. We found everything in efficient working, the patients supremely happy, and the situation and accommodation as good as possible. We noticed, as we have said, that the patients mostly suffered from open wounds, and that these semi-convalescent hospitals are not used, as we expected to find them, for the reception of cases after operations who have arrived at the stage where the general health of the patient is not proceeding as rapidly as desirable, though the wounds may be healed. Adjacent to the semi-convalescent hospitals is an ordinary convalescent hospital, the Cookridge, which contains 150 beds, and is open for nine months in the year. This is largely used by the patients from the General Infirmary, and it is the practice to pass the patients from the semi-convalescent hospital beds as rapidly as possible by moving them into the adjacent convalescent home, whenever practicable.

### Badly-planned Hospital Buildings.

One remarkable fact, now historical, well illustrates how little medical and hygienic authorities recognised the importance of superficial area and abundance of air and light in 1864, when the General Infirmary plans were accepted. Emphasis is given to this important point by the circumstance that the architect selected was no less a person than Sir Gilbert Scott, who, as may be anticipated, adopted the Gothic form to the fullest possible extent. The Gothic is not well adapted for hospital purposes, and much of the limited space in the lavatories and elsewhere may be put down to this feature of the buildings. The fact referred to is that an ample site was originally purchased, but, strange to say, instead of devoting the whole of it to hospital purposes, which was essential, as will be seen a little later on, if full force was to be given to hygienic considerations, the authorities of those days disposed of £15,000 worth of ground which they let out as surplus land. The want of apprehension which led to this initial mistake was further shown by the managers in subsequent years, who, instead of remedying the patent defects of the existing buildings by utilising some available space at the back of the main buildings, rendered all such hygienic and necessary reconstruction impossible, so far as the present site is concerned, by placing upon this vacant space a nurses' home. The grave error which was thus committed becomes at once apparent on entering the hospital and making an inspection of the ground floor. It is scarcely credible, but it is the fact, that on this floor have been placed (1) the kitchen, (2) an unusually large laundry, and (3) the dead-house and post-mortem rooms. It would be bad enough to have these departments where they now stand if precautions had been taken to prevent any emanation from them penetrating to the centre of the building. Instead of this being done, the noises, the smells, and the bad air originating in these departments must necessarily reach the wards, for every one of them opens on to the corridor from which staircases and lifts ascend to each of the ward pavilions. We call special attention to these hygienic errors in justice to the present managers, whose difficulties must necessarily be increased by these grave defects in the original plan of construction, which have handicapped this hospital for forty years. In the present day it often happens that the rush and thirst for new buildings leads to an enormous waste of valuable funds. We do not, however, remember to have met with a much worse instance of how not to do it than is exhibited in the respects mentioned by the original plans of this infirmary.

A defect which must render the work onerous compared with that in more modern hospitals is the fact that the ward units are not complete, for they do not include some of the rooms and adjuncts now found essential for the ready and perfect administration of a large hospital ward. Another interesting point in the construction of this hospital, as indicating the stage of development reached by the hospitals of forty years ago, is that no nursing home was thought of or provided in the original plan. When, however, a home was at length thought necessary, a relatively small building was erected on the site where the laundry, dead-house and kitchen might have stood. It soon became necessary to double the size of the nursing home. When this was done the then treasurer believed that it would be too large, if anything, for its purpose. To-day the nurses' home has extended by means of a tunnel across the street into a large and well-equipped home, which has already proved far too small, and the managers have before them at the moment the problem of building yet another and larger building still for the accommodation of nurses. There is no error so costly in hospital construction as the initial error of making the new buildings and sections of buildings too small, save it be the failure at the outset to provide, when selecting town sites, an abundant reserve of land for future extensions.

### The Present Condition of the Hospital.

We had the advantage of being accompanied by the Treasurer, Mr. Charles Lupton, whose able administration under most difficult circumstances of the affairs of the Leeds General Infirmary does him honour. He devotes a large portion of his time to the work, and is one of the world's examples of the men who from conviction give much continuous thought and individuality to the work of making adequate provision for the sick in the community amongst whom they reside. Mr. Lupton is in want of a good deal of money for the infirmary, and it ought to be given him by a grateful public, for he has shown, as the books and accounts and system of management at Leeds to-day prove, that he is one of the most economical and careful managers to be met with amongst British hospital officials. The administration of this hospital is handicapped by a feature of the buildings which should have formed no part of the plan, and would not be tolerated to-day. What ought to have been a central courtyard open to the air, with the ward pavilions on either side of it, was for some unexplained reason covered in with a roof which serves the purpose of restricting the free circulation of air around and about several ward blocks. The covered space was originally intended as a winter garden for the use of convalescent patients, but proving a failure in this respect it was partly occupied as a carpenter's workshop and store, and has at present a lawn tennis court in the centre of it. If hygienic considerations are to prevail, the best thing to do with it would be to sweep it away entirely and to let in the air and daylight which are at present kept out of the wards.

### In the Wards.

The administration of the wards is rendered, in our judgment, more than usually difficult by the fact that many of the wards contain 32 beds, a number not easy to administer or to nurse. They are well kept, having regard to the number of years they have been in use, and we found most of them in good order. Many of the fittings are out of date, and they are being gradually replaced by more modern appliances. Excellent work is done in these wards, but the pressure upon the beds and staff is great, and it appears to us that overwork is the rule rather than the exception. This is especially the case in the surgical wards, which bore evidence of the rush and stress of the work and the want of more nurses in the larger wards. The need for more nurses may be exhibited by pointing out that compared with the Royal Infirmary, Liverpool, a most efficiently administered institution, the present staff of sisters and nurses, which amounts to 102, should be increased to 115 by the addition of three sisters and ten nurses and probationers. The Leeds scheme of nursing differs from that of most hospitals in the fact that it employs more trained nurses and many fewer probationers than is the rule at other similar institutions. Thus Leeds has 11 more nurses and 21 fewer probationers in proportion to the number of beds than prevails at Liverpool. Another disadvantage under which this hospital labours administratively is the limited number of operation theatres and the extreme pressure which exists upon those theatres practically every day in the week. This is a point which every hospital committee ought to consider in the most liberal spirit, because where the theatre is in use for many consecutive hours it must not be forgotten that, although the surgeons engaged may change two or three times in the course of the day's work, the sisters and nurses have, under existing conditions, too frequently to remain on duty for too many hours. We say too many hours because the temperature of the theatre is often high, the condition of its atmosphere after several hours affects the condition of those who remain too long in it, and, where the same staff has to be in the theatre every day in the week, the strain is calculated to affect the health of the most robust and healthy men and women. It is well known to matrons and lady superintendents that great care has to be taken to pick out the most healthy members of the staff for this branch of work, a fact which we commend to the consideration of every hospital committee in the country.

### Nurses' Training.

The inspection we made of the hospital afforded ample evidence of the efficiency of the training which the nurses receive here under somewhat onerous and difficult conditions. We were, for instance, greatly pleased when we found that the sister-in-charge of one ward, which was in the most perfect order throughout, had been trained at Leeds within the last five years. No better testimony to the thoroughness of Miss Fisher, who has rendered such invaluable service to this infirmary, could be afforded. We were glad to notice that wherever possible Mr. Lupton and his colleagues have endeavoured to meet modern requirements. This is especially testified by the construction of a balcony for the children, into which cots can be wheeled out. It has a sunny aspect—a rather difficult condition in regard to balconies in a hospital planned like the General Infirmary at Leeds. We found a mortuary here sufficient but crude in its arrangements. As we have said many times, we think a great hospital owes it to its reputation to enforce reverence for the dead in justice to the living. We should like to see the provision of a mortuary chapel in which the dead might be placed, and where the friends could see them when necessary. In any case, it is far better in our judgment to do away with the rods and curtains which divide the mortuary chamber into spaces, and to substitute for them iron couches, as is done in most of the best administered hospitals at the present day.

Speaking generally, we should say that the time is approaching when it will be desirable, if it is not even necessary, to reconsider the whole plan of the existing buildings, and to decide whether the rebuilding shall take place on the present site, or whether, as in the case of Manchester, the hospital shall be moved out from the centre to a more airy and more suitable site.

### Sound Administration.

We should say of Leeds, as we have felt for many years, that on the whole, if the management errs at all, it errs on the side of economy. Great care is taken to prevent the abuse of the hospital by those who are not proper recipients of its benefits. In the purchase and distribution of the stores everything has been done to secure a pound's value for every pound expended. In the internal administration and working of the hospital strict supervision is exercised over the expenditure under all heads. By an elaborate system which has been perfected since Mr. Lupton became treasurer, a monthly report is made, giving the approximate cost of occupied beds under the heads of provisions, wines, spirits, ale, stout, aerated waters, new milk, drugs and sundries, dressings, glass urinals, clinical thermometers, glass and earthenware, hardware, floor polish, brushes and baskets, nurses' uniforms, nurses' salaries, wardmaids' wages, special outside nurses, massage, commissionaires, indiarubber goods, glass, plates, papers, etc., with the total cost under all heads for each ward and department, including the out-patients', casualties, theatres, old in-patients', pathological, *x*-ray and Finsen light departments. This return has been most fruitful in results. It reveals to the sister of each ward and to the honorary and resident staff who are responsible for its efficient working exactly how it compares with its neighbours, and a friendly competition in economy has been created which has no doubt helped materially to make the average cost per occupied bed at this hospital below the average of most hospitals of its size. We hope to find room for a specimen of this return in a future issue, for it is one which we commend to the earnest consideration of every hospital where the authorities are anxious to secure full economy with the maximum of efficiency. In any case, as we have already stated, anyone in Leeds who has money to spare may, with the fullest confidence, place it, or some of it, at the disposal of the treasurer and committee of the Leeds General Infirmary. Here money is badly wanted, and will be well applied for the most beneficial of public purposes, the speedy restoration of the disabled worker to a position in which he can make his earning power felt to the full for the benefit of his family and those dependent upon him.

Some **Welsh** and **Cottage Hospitals** will be reported on next week.

Literary Notes.—In the *British Journal of Tuberculosis* for January, which is well illustrated as usual, Dr. Kirkpatrick, of Steevens' Hospital, Dublin, deals with Irish writers on tuberculosis in the eighteenth century. The liveliest of these seems to have been one Thomas Marryat, with his "The New Practice of Physick, founded on Irrefragable Principles, and confirmed by Long and Painful Experience." His own painful experience was happily short, for on a diet of milk and broths—"especially of pork (by the use of which I've seen miracles perform'd)"—he got well of consumption in six weeks. This writer also advises the eating of snails, and the opinion has been heard in quite recent times. It is probably an instance of the doctrine of signatures, a snail's body resembling phthisical sputum fairly closely both in colour and consistence. In the same number Dr. G. H. R. Dabbs has a short paper on "Genius and Tuberculosis." Dr. Dabbs's reputation for verse is well earned; but this specimen, at any rate, of his prose recalls somewhat the soaring diction of Mr. Putnam Smif in "Martin Chuzzlewit."

